# How to manage hypersensitivity reactions to enzyme replacement therapy in lysosomal storage diseases?

**DOI:** 10.1186/s13023-025-03844-8

**Published:** 2025-06-06

**Authors:** Federico Spataro, Antonio Giovanni Solimando, Vanessa Desantis, Angelo Vacca, Attilio Di Girolamo, Roberto Ria

**Affiliations:** 1 Post Graduate School in Allergology and Internal Medicine “Guido Baccelli”, Department of Precision and Regenerative Medicine and Ionian Area—(DiMePRe-J), School of Medicine, University of Bari Aldo Moro, Piazza Giulio Cesare, 11, Bari, 70124 Italy; 2 Guido Baccelli Unit of Internal Medicine, Department of Precision and Regenerative Medicine and Ionian Area—(DiMePRe-J), School of Medicine, Aldo Moro University of Bari, Bari, 70124 Italy; 3 Department of Precision and Regenerative Medicine and Ionian Area - DiMePRe-J, Section of Pharmacology, School of Medicine, University of Bari Aldo Moro, Bari, 70124 Italy

**Keywords:** Drug allergy, Enzyme replacement therapy, Lysosomal storage diseases, Desensitization, Omalizumab

## Abstract

Lysosomal Storage Diseases (LSDs) encompass a range of genetic disorders characterized by enzyme deficiencies that lead to substrate accumulation and progressive tissue damage. Enzyme Replacement Therapy (ERT) is the primary treatment for LSDs, yet it is often associated with hypersensitivity reactions (HSRs), ranging from mild rashes to severe anaphylaxis. These reactions, frequently driven by anti-drug antibodies, pose significant challenges in treatment adherence and patient outcomes. This paper outlines a stepwise approach to managing HSRs during ERT, focusing on three escalating strategies. The first-line approach involves premedication using antihistamines, corticosteroids, antileukotrienes, and bronchodilators to prevent or reduce the severity of HSRs.

For patients who continue to experience HSRs despite premedication, desensitization protocols are recommended, involving the gradual reintroduction of ERT in controlled, increasing doses.

In cases of refractory HSRs, omalizumab, a monoclonal antibody targeting IgE, has been successfully used as a third-line intervention.

This comprehensive, stepwise strategy aims to provide clinicians a guide to manage these challenges, offering practical steps for optimizing treatment while ensuring patient safety. Future research is needed to further validate these management techniques and explore new therapeutic options for optimizing care in this rare but critical patient population.

## Introduction

Lysosomal Storage Diseases (LSDs) encompass a heterogeneous group of about 70 rare genetic disorders characterized by the deficiency of specific enzymes responsible for the degradation of substrates within the lysosomes. The progressive accumulation of these substrates leads to widespread cellular and tissue damage, manifesting in diverse clinical features ranging from mild to life-threatening conditions. Prominent examples of LSDs include Gaucher disease, Fabry disease, Pompe disease, and Mucopolysaccharidosis (MPS) [1].

Enzyme Replacement Therapy (ERT) is the cornerstone of treatment for several LSDs, aimed at supplementing the deficient enzyme and preventing further substrate accumulation [1, 2]. Despite its life-saving potential, ERT is frequently associated with hypersensitivity reactions (HSRs), which significantly compromise treatment adherence and patient outcomes. HSRs during ERT can range from mild symptoms such as rash, urticaria, cough and pruritus, to severe anaphylaxis. The incidence of these reactions varies, with studies reporting HSRs in up to 20% of patients receiving ERT [3, 4]. Currently, no specific demographic factors, such as age, sex, or genetic predisposition, have been scientifically established as increasing the risk of HSRs to ERT.

These reactions are often mediated by anti-drug antibodies, IgG or IgE, which can trigger immune responses. The management of HSRs is critical for ensuring continued access to ERT and optimizing patient outcomes. In this communication, we explore a stepwise approach to managing HSRs in LSD patients, from first-line strategies to advanced interventions.

## Mechanisms of hypersensitivity reactions

ERT-related hypersensitivity reactions are predominantly Type I (immediate) reactions, with different mechanisms that can contribute to these adverse events.

Many HSRs to ERT in LSD patients may have an IgE-mediated mechanism that occur when specific anti-recombinant-enzyme IgE antibodies bind to high-affinity IgE receptors (FcεRI) on the surface of mast cells and basophils. Upon re-exposure to the drug, these antibodies cross-link, leading to cell degranulation and the release of preformed mediators such as histamine and tryptase. The clinical manifestations of this process include pruritus, flushing, urticaria, angioedema, throat tightness, shortness of breath, and, in severe cases, hypotension and cardiovascular collapse.

However, these cells can be activated independently of IgE through the Mas-related G protein-coupled receptor X2 (MRGPRX2). Drugs can bind directly to MRGPRX2 on mast cells and basophils, leading to non-IgE-mediated degranulation, producing similar symptoms to those of IgE-mediated reactions. This alternative pathway is particularly relevant for reactions that occur during the first exposure to a drug, where no prior sensitization has occurred.

Another critical pathway involves the activation of the complement cascade. Certain drugs can trigger the complement system, resulting in the release of C3a and C5a anaphylatoxins. These anaphylatoxins bind to their respective receptors on mast cells and basophils, causing degranulation and the release of histamine and tryptase. This leads to a similar clinical picture as IgE-mediated reactions, with symptoms such as urticaria, angioedema, and cardiovascular compromise.

Moreover, other cells such as monocytes, macrophages, and T cells may also play a role in HSRs associated with ERT. These cells release pro-inflammatory cytokines like IL-6, TNF-α, and IL-1β, which contribute to systemic symptoms including fever, chills, hypotension, nausea, vomiting, fatigue, dyspnea, and oxygen desaturation. These reactions, often classified as infusion-related reactions (IRRs), are more common during initial exposures and are typically milder but can become severe in some patients [5, 6].

The release of these cytokines induces systemic inflammatory responses that manifest as flu-like symptoms, including fever, fatigue, and myalgias, as well as more serious complications like hypotension and oxygen desaturation, which can mimic anaphylaxis.

Skin tests (prick and intradermal tests) should always be performed after an adverse reaction to ERT to investigate the underlying mechanism: an IgE-mediated reaction if positive, or a non-IgE-mediated reaction if negative. Generally, for recombinant enzymes, skin prick test is performed with the undiluted drug, followed by intradermal testing with 1/100 and 1/10 dilutions. However, data on their sensitivity for these drugs are limited, as no studies have established non-irritant testing concentrations [6]. When available, it is advisable to complement skin testing with serum assays for specific anti-drug IgE or IgG antibodies.

## First line approach: premedication and/or slow infusion rate

Premedication is the initial strategy to prevent or mitigate HSRs in patients undergoing ERT. The choice of premedication depends on the patient’s history, type and the severity of previous reactions. Patients, based on the symptoms and clinical manifestations of the adverse reaction, may benefit from one or more of the following premedication drugs. Below, the rationale for the use of each medication:

- *Antihistamines*. H1 and H2 antihistamines, such as diphenhydramine and ranitidine, are frequently administered to block histamine receptors and reduce the risk of urticaria, angioedema, and pruritus during ERT. How to use: 30 min before the infusion [5].
- *Corticosteroids*. Glucocorticoids are often added for patients with moderate to severe HSRs. They act by suppressing immune activation and reducing the severity of inflammatory responses. Anyway, their prolonged use may lead to important side effects [7]. How to use: 30–60 min before the infusion.
- *Antileukotrienes*. Montelukast, a leukotriene receptor antagonist, is used particularly in patients who experienced cough, bronchospasm or asthmatic symptoms during ERT [5, 8]. How to use: from two days before the infusion.
- *Non-Steroidal Anti-Inflammatory Drugs (NSAIDs) and paracetamol*. Evidence shows that NSAIDs (e.g., aspirin) can be employed to manage flushing by blocking the arachidonic acid pathway and mitigating vasodilation. They have been successfully used as premedication to minimize flushing, including in the pediatric population experiencing HSRs to ERT. On the other hand, paracetamol can be used for fever and myalgia [6, 9]. How to use: 30 min before the infusion.
- *Bronchodilators*. Beta-agonists (e.g., formoterol) may be indicated for patients with a history of airway hyperreactivity or asthma triggered during ERT [10]. How to use: 5–10 min before the infusion.

The combination of these agents is tailored to each patient’s risk profile, often allowing continued administration of ERT with minimized adverse effects.

Moreover, for many mild adverse reactions, it has been observed that reducing the infusion rate from the initial steps would make the drug more tolerable.

## Second line approach: desensitization

For patients who experience recurrent HSRs despite adequate premedication or slowing the infusion rate, desensitization represents the next therapeutic step. It should be considered an add-on strategy rather than a replacement for premedication. Desensitization involves the gradual reintroduction of the enzyme in controlled, increasing doses, allowing the immune system to tolerate the allergen temporarily.

The classic procedure is the *rapid desensitization* approach that entails the induction of a temporary tolerance to ERT by administering the offending drug with increasing dosages over a longer period compared with standard infusion schedule, up until the full cumulative therapeutic dose is given and tolerated. Usually, it lasts about 6 h and include 12 consecutive steps using three bags of solutions with increasing drug concentrations (Table 1). Rapid desensitization is indicated both for patients with Type I HSRs including IgE-mediated reactions and non IgE-mediated reactions. Evidence on the mechanism of rapid drug desensitization is still challenging. However, mast cells and basophils seem to be the main actors of this procedure because mediators from these cells are released during drug-induced hypersensitivity responses. Nevertheless, the first hypothesis is that increasing sub-therapeutics dosages of the antigens bind to IgE anchored to the surface FcεRI, but the cross-linking does not occur. Alternatively, the antigen might trigger the swift internalization of cross-linked antigen receptors, reducing their presence on the cell surface and rendering the cell unresponsive to the antigen [10, 11]. During the initial infusions under rapid desensitization, clinicians should combine premedication with desensitization. If the procedure is well tolerated, premedication can be gradually reduced, followed by a stepwise decrease in infusion time under desensitization, aiming to return to the standard infusion protocol.

As with any medication, the prescribing information outlines the maximum infusion rate, potential instability from excessive dilution, and the allowable time frame for administration after reconstitution. Clinicians must take these factors into account when managing infusions. While these drugs are typically not prepared under a laminar flow hood, performing dilutions in such conditions is recommended to reduce the risk of contamination and infection, particularly considering the inherent fragility of these patients.

On the other hand, *AIT-like desensitization*, a novel approach inspired by allergen immunotherapy (AIT) used in Hymenoptera venom allergies, has been successfully attempted in MPS patients with IgE-mediated HSRs, as both ERT and Hymenoptera venom share enzymatic allergenic components. It consists of the gradual subcutaneous administration of recombinant enzymes with the aim of conferring long-term immune tolerance. The mechanisms involved in the AIT-like desensitization procedures consist of the activation of T-regulatory cells with the production of IL-10, TGF-β, and IL-35, which switch off the allergy response by downregulating B-cells, mast cells, basophils [12]. The difference between rapid desensitization and AIT-like desensitization is that a long-lasting tolerance could be achieved only with the second one. This statement is further supported by our recently published experience with three MPS patients who successfully returned to the standard infusion protocol within a short timeframe using this desensitization approach. Notably, one patient (Patient 3) who had previously tested positive on skin tests for idursulfase showed negative skin test results one year after the combined desensitization approach, indicating the achievement of complete tolerance [13]. However, AIT-like desensitization is not yet widely adopted, with only one other reported case involving a different drug, trastuzumab [14].

## Third line approach: omalizumab

Omalizumab, a monoclonal antibody targeting IgE, administered subcutaneously and approved for allergic asthma, chronic spontaneous urticaria (CSU), and nasal polyps, acts by binding circulating IgE, preventing it from interacting with FcεRI on mast cells and basophils, thereby reducing allergic responses and mast cell degranulation. Omalizumab has already been used successfully to manage HSRs to chemotherapy (e.g. platins, taxanes), in both IgE-mediated and non-IgE-mediated reactions [15, 16]. While the mechanism of action of omalizumab in IgE-mediated reactions is well understood—given its role in blocking specific IgE that could potentially trigger an allergic response—its mechanism in non-IgE-mediated reactions remains unclear despite documented efficacy. A notable example is CSU, where omalizumab has been shown to be effective despite the absence of an IgE-mediated cascade. In CSU, although the precise mechanism of action is not fully elucidated, it is hypothesized that omalizumab reduces free IgE levels and downregulates FcεRI expression on dermal cells, both in lesional and non-lesional skin, as well as on basophils, thereby decreasing cellular responsiveness [17].

In the context of LSDs, omalizumab has been used as a valuable adjunct to desensitization in patients who experience severe or refractory HSRs to ERT [15]. In the literature, there are three case reports demonstrating the successful use of omalizumab in LSD patients.

DuBuske et al. reported a case of a 38-y-o. man with Fabry disease who experienced severe anaphylaxis during ERT and tested positive for skin test to the recombinant enzyme. Given the poor results obtained with rapid desensitization, the introduction of omalizumab 300 mg monthly as premedication allowed the resumption of ERT without further HSRs [18].

In the cases described by Arroabarren et al. and Diaz Vidal et al., omalizumab was successfully used to manage refractory hypersensitivity reactions in two patients with MPS type IV-A when desensitization protocols failed. The first patient, a 5-year-old boy with positive skin tests to the recombinant enzyme, was treated with omalizumab at 75 mg monthly, while the second patient, a 22-year-old woman with negative skin tests, received omalizumab at 300 mg every two weeks [19, 20].

Finally, in our recent study (Spataro et al., 2024) an adult male with MPS type I-S (Patient 2), who developed HSR to laronidase, was initially managed with a combined desensitization approach. Despite successfully tolerating standard infusions for 13 months, the patient later experienced a severe HR. A second desensitization attempt was unsucessfull. At this point, omalizumab 300 mg monthly was introduced as an adjunctive therapy. This allowed the patient to resume immediately laronidase infusions without further reactions. After six months, omalizumab dosing was tapered to 150 mg monthly, maintaining tolerance [13].

These cases highlight the significant potential of omalizumab as a third-line option for managing HSRs in LSD patients who continue to experience such allergic reactions despite the use of premedication and desensitization protocols. Importantly, this approach has proven effective irrespective of the patient’s skin test results to ERT, demonstrating its utility in both IgE-mediated and non-IgE-mediated reactions. This suggests that omalizumab can play a crucial role in cases where conventional management strategies have failed, offering a versatile solution even when the underlying immunological mechanism remains unclear. Furthermore, in cases where omalizumab demonstrates beneficial effects, the dosage could be reduced by half after six months of tolerance to ERT, with the possibility of eventual discontinuation thereafter.

## Discussion and conclusion

Managing hypersensitivity reactions in lysosomal storage diseases presents a complex challenge, given the life-saving role of enzyme replacement therapy. The stepwise approach outlined in this paper emphasizes a graduated escalation of interventions, beginning with premedication and advancing through desensitization and omalizumab, depending on the severity and recurrence of HSRs.

Premedication remains the first-line strategy, but its effectiveness is often limited in patients with mild to moderate reactions. On the other hand, when HSRs are severe or not responsive to premedication, desensitization offers a viable alternative by modulating the immune response and allowing continued treatment. Rapid desensitization has been widely adopted, but recent advances in AIT-like approaches for IgE-mediated HSRs show promise in achieving longer-term tolerance.

Omalizumab represents a crucial addition to desensitization for patients with refractory HSRs, particularly when the late strategies have been exhausted. The cases reported in the literature provide compelling evidence for its use in this specific context, expanding its application beyond its approved indications in allergic asthma and chronic urticaria. Another challenge would be to determine how to manage long-term omalizumab treatment in terms of dosing, specifically whether it can be tapered over time and what the appropriate timelines for such tapering would be.

There are currently no available genetic profiles or predictive biomarkers for the development of allergic reactions to ERT. However, what can be done is to identify patients at higher risk of reactions during desensitization (also called breaktrough reactions) and, consequently, predict the likelihood of success for this procedure. In particular, patients with negative skin tests are at lower risk, while those taking beta-blockers or ACE inhibitors are at higher risk, as are individuals with underlying cardiac conditions, asthma, or mastocytosis [21].

**Fig. 1:**
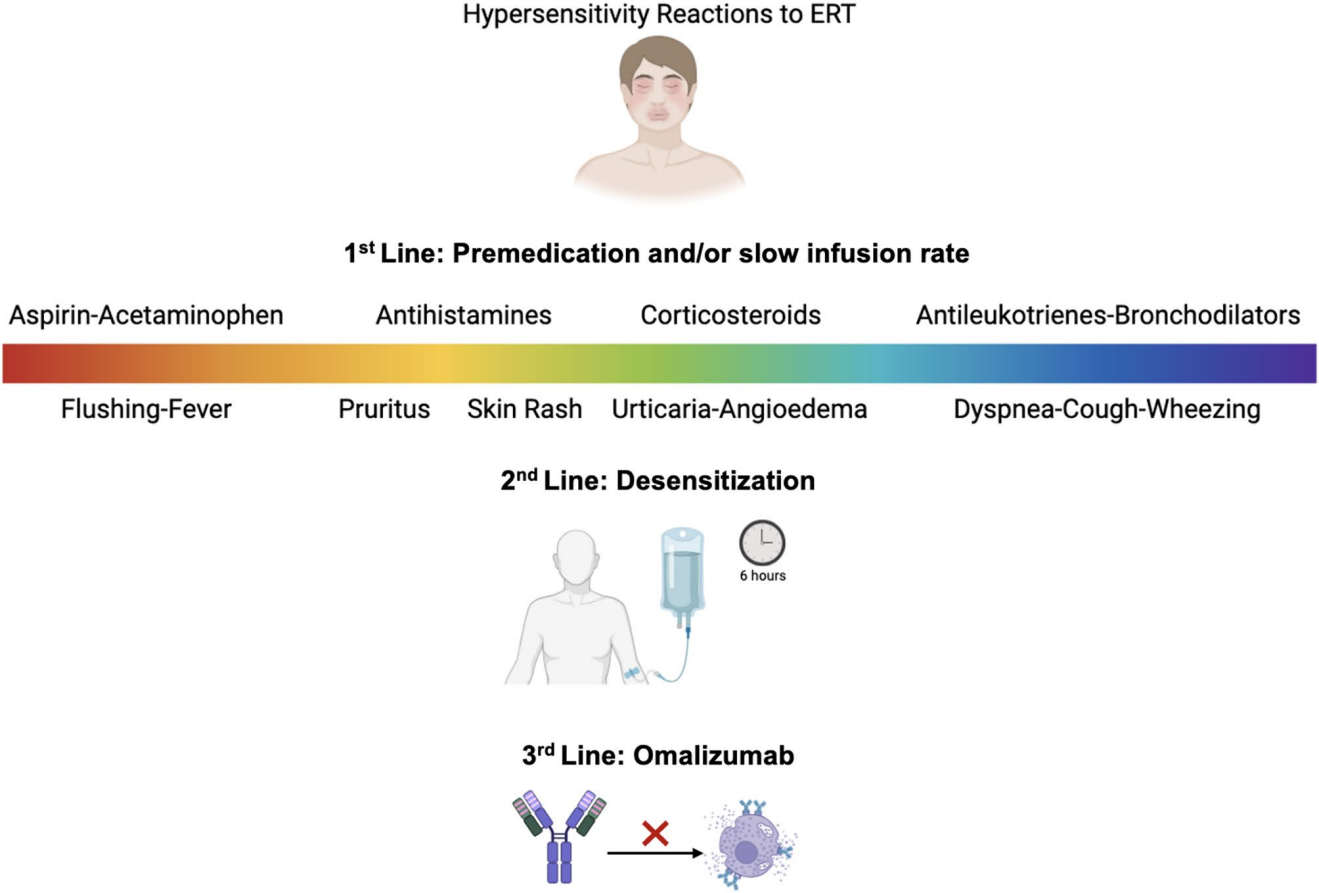
Management of hypersensitivity reactions to enzyme replacement therapy in lysosomal storage diseases. ERT, enzyme replacement therapy

In conclusion, the management of HSRs in LSD patients requires a personalized, multi-step approach as depicted in Fig. 1, with the integration of premedication, desensitization, and omalizumab as needed. Future studies are essential to further validate these strategies and explore novel therapeutic avenues to optimize care for this vulnerable patient population.

**Table 1:** Rapid desensitization for idursulfase

step	solution	minutes	rate (ml/h)	delivered ml	delivered dose (mg)
1	1/100	15	4	1	0.00048
2	1/100	15	10	2.5	0.0012
3	1/100	15	20	5	0.0024
4	1/100	15	40	10	0.0048
5	1/10	15	10	2.5	0.012
6	1/10	15	20	5	0.024
7	1/10	15	40	10	0.048
8	1/10	15	80	20	0.096
9	1/1	15	20	5	0.24
10	1/1	15	40	10	0.48
11	1/1	15	80	20	0.96
12	1/1	182	150	455	21.84
total		347			23.71

Target dose: 24 mg. Dilution 1/100 (bag 1): 10 ml of dilution 1/10-bag 2 in 90 ml of saline (0.00048 mg/ml). Dilution 1/10 (bag 2): 10 ml of dilution 1/1-bag 3 in 90 ml of saline (0.0048 mg/ml). Dilution 1/1 (mother solution; bag 3): 24 mg of idursulfase in 500 ml of saline (0.048 mg/ml)

## Data Availability

Data related to this manuscript will be made available upon request.

## Abbreviations

AIT: Allergen immunotherapy
CSU: Chronic spontaneous urticaria
ERT: Enzyme replacement therapy
HSRs: Hypersensitivity reactions
LSDs: Lysosomal storage diseases
MPS: Mucopolysaccharidosis
